# Unexpected Favorable Outcome to PD-1 Antibody Plus Lenvatinib in a Patient With Recurrent Intestinal Follicular Dendritic Cell Sarcoma: A Case Report and Literature Review

**DOI:** 10.3389/fimmu.2021.653319

**Published:** 2021-09-08

**Authors:** Yanna Lei, Sha Zhao, Ming Jiang

**Affiliations:** ^1^Center of Medical Oncology, West China Hospital, Sichuan University, Chengdu, China; ^2^Department of Pathology, West China Hospital, Sichuan University, Chengdu, China

**Keywords:** follicular dendritic cell sarcoma, immune checkpoint inhibitor, sintilimab, lenvatinib, antiangiogenesis

## Abstract

**Background:**

Follicular dendritic cell sarcoma (FDCS) is an uncommon malignant cancer, and there is no standard treatment to date. Resection followed by adjuvant chemotherapy or radiation is considered the most commonly used strategy for treatment. However, the treatment for patients who have progressed after systemic treatment is more controversial.

**Case summary:**

In this case report, we describe a 57-year-old man with primary small intestine FDCS where surgery and second-line systemic chemotherapy failed. After disease progression (PD), the patient received sintilimab plus lenvatinib as third-line treatment and achieved a progression-free survival (PFS) with 7 months.

**Conclusion:**

This is the first report of a FDCS patient treated with immune checkpoint inhibitors (ICIs) and antiangiogenic agents, sintilimab and lenvatinib, as third-line therapy. Our case provides a potential therapeutic option for patients with FDCS who progressed after multiline therapy.

## Introduction

Follicular dendritic cell sarcoma (FDCS) is a rare hematologic cancer that usually arises in lymph nodes and in extranodal sites, including liver, lung and head-and-neck soft tissue (1). Fewer than 1000 cases have been reported in the literature, and very few cases of primary small intestinal FDCS have been described thus far (2).

The combination of ICI and antiangiogenic therapy results in dramatic tumor reduction in several solid cancers, such as hepatocellular carcinoma (3) and renal cell cancer (4). However, no case report described such a treatment strategy in FDCS patients who progressed after multiline therapy. Here, we present a case study of recurrent FDCS that responded remarkably to the combination of sintilimab and lenvatinib. The PFS of third-line treatment reached 7 months, while the PFS of second-line treatment was 3 months. Furthermore, we discuss the rationality of such therapy based on published research. Previous reports on the treatment of FDCS are also briefly reviewed in this report.

## Case Report

**Figure 1:**
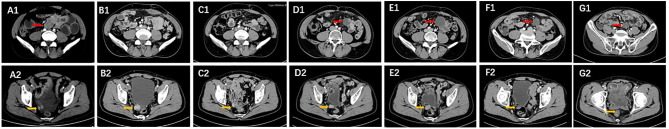
The lesions indicated by arrows at different time points. **(A1, A2)** Lesions were observed before surgery in the CT images in April 2019. **(B1, B2)** Images after surgery in May 2019. **(C1, C2)** Partial remission of the lesions after two cycles of CHOP. **(D1, D2)** New lesions can be seen in March 2020 after a PFS of 2 months. **(E1, E2)** The lesions were expanded after two cycles of ABVD chemotherapy. **(F1, F2)** The lesions shrunk after 3 cycles of sintilimab and lenvatinib. **(G1, G2)** Partial remission of the lesions were shown after 6 cycles.

**Figure 2:**
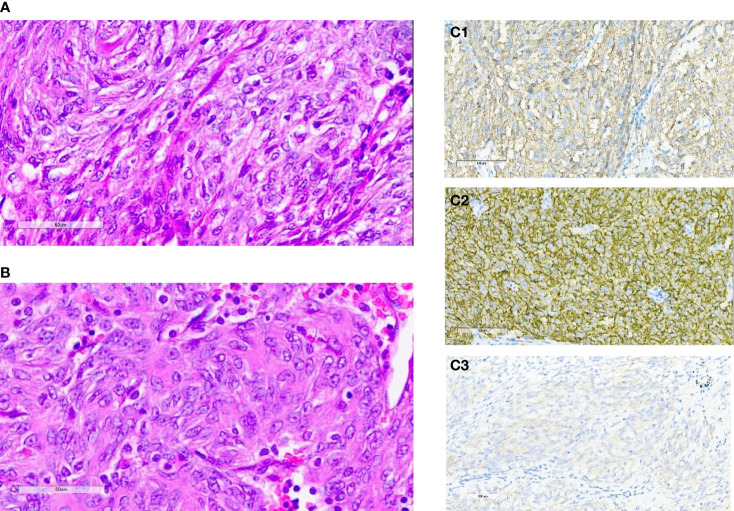
**(A, B)** Hematoxylin-eosin staining of primary tumors: small intestinal spindle cell tumors. **(C)** Immunohistochemical staining for CD21 **(C1)**, CD23 **(C2)** and CD35 **(C3)**.

**Figure 3:**
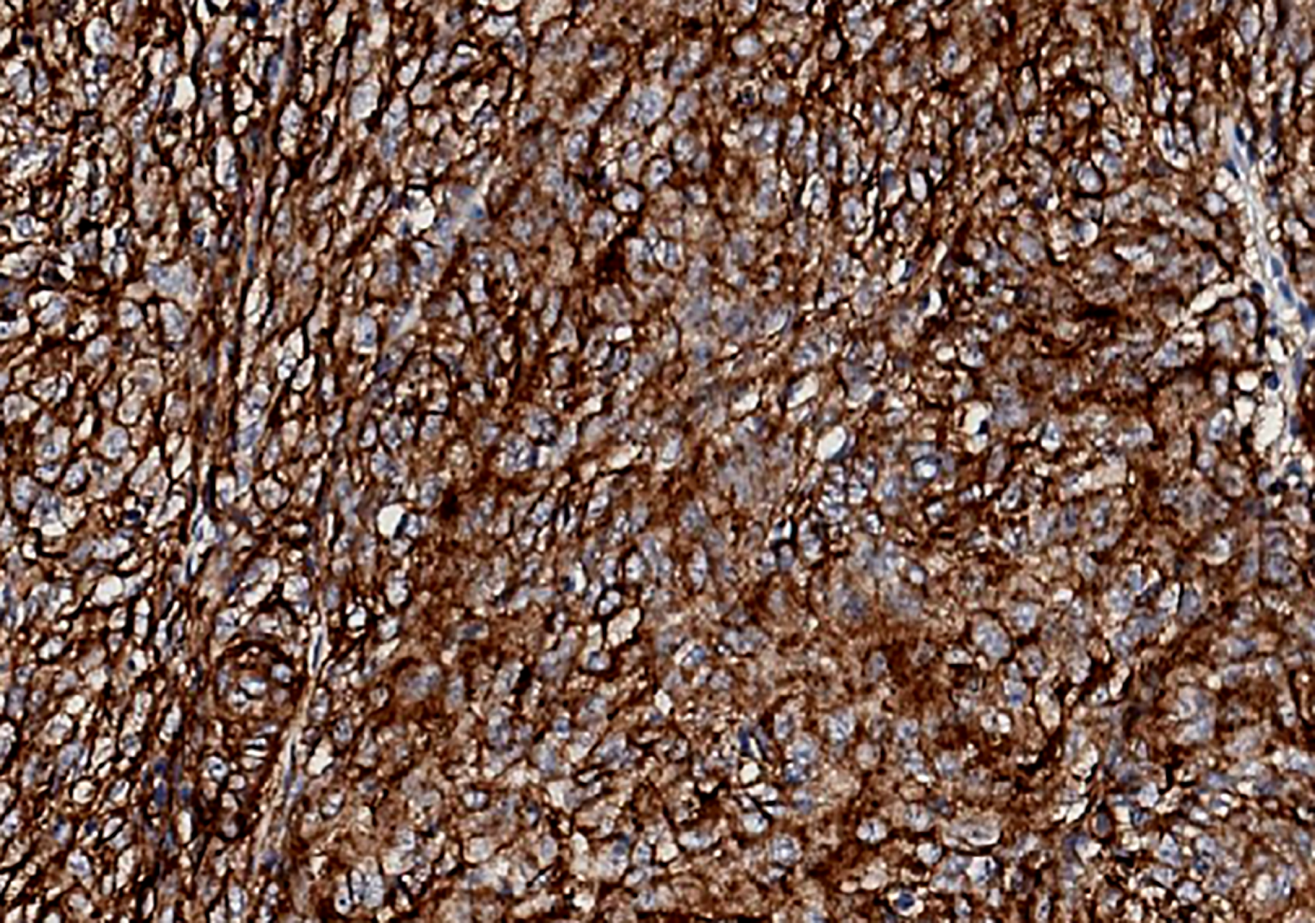
Immunohistochemical staining of tumor cells for PD-LI (22C3) in our patient. PD-L1 expression was 90%.

**Figure 4:**
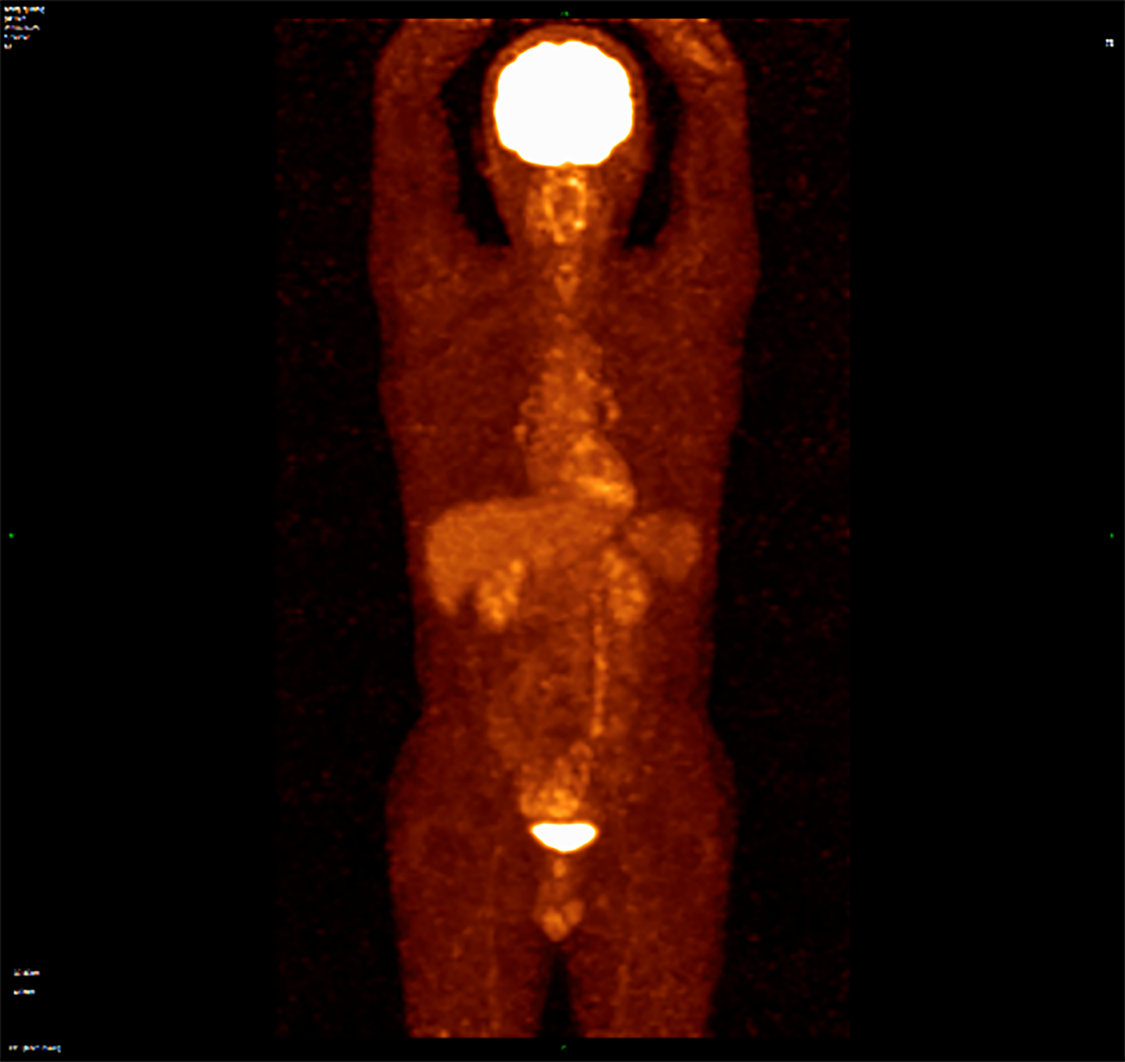
PET/CT did not show focal hypermetabolism of those lesions corresponding to the nodules in CT.

A 57-year-old Chinese man presented to our hospital with acute left lower quadrant abdominal pain in April 2019. He denied dyspnea, fever, night sweats, and weight loss. He is a non-smoker and a teetotaller. He denied any personal or family history of cancer. Physical examination was unremarkable, and his Eastern Cooperative Oncology Group (ECOG) performance status (PS) was 1. An abdominal computed tomography (CT) scan showed an 8.9 ×5.7 cm solid mass in the left abdominal cavity (**Figure 1A1**), and many soft tissue density shadows were observed in the abdominal cavity, especially in the rectovesical pouch (**Figure 1A2**). Partial intestinal resection was performed with a mechanical side-to-side anastomosis and negative margins were confirmed intraoperatively in April 2019.The pathological result after the operation was as follows: small intestinal spindle cell tumor (**Figures 2A, B** **)**. Combined with morphological and immunohistochemical results, follicular dendritic cell sarcoma was diagnosed pathologically. Multiple tumor nodules were found in mesentery. Immunohistochemistry (**Figure 2C**): tumor cells CD21 (+, partial), CD23 (+ partial), CD35 (weak positive), sstr-2 (+, partial), CD163 (+ partial), cd68/pgm-1 (+, focal area), HMB45 (–), PCK (–), ALK (–), LCA (–), EMA (–), STAT-6 (–), syn (–), TLE (–), DOG-1 (–), SDHB (without delated), CD117 (–), DES (–), CD34(-), SMA (–), S100 (–), Ki-67 (+, 10% - 20%), *in situ* hybridization EBER1/2 (–), PD-1 (–), and PD-L1 (+90%) (**Figure 3**). Next-generation sequencing (NGS) was applied in the specimen of the patient, but result of targeted genetic testing was negative. The CT after surgery showed multiple nodules in the mesentery, pelvic cavity and rectovesical pouch, and the largest nodule was 2.5*2.2 cm located in the rectovesical pouch (**Figure 1B**). However, PET/CT did not show focal hypermetabolism of those lesions corresponding to the nodules in CT (**Figure 4**). The laboratory tests, including complete blood count, liver function, renal function, serum lactate dehydrogenase level and cardiac function, were in normal limits.

**Figure 5:**
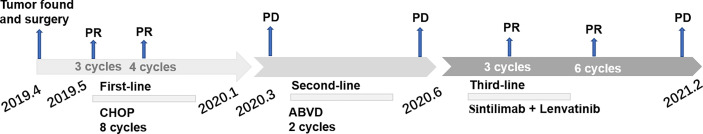
Timeline scheme of major clinical event of the patient since diagnosis.

The patient received eight cycles of CHOP chemotherapy (Cyclophosphamide 750 mg/m^2^ d1, Doxorubicine 50 mg/m^2^ d1, Vincristine 1.4 mg/m^2^ d1 and Prednisone 100mg d1-5 every 3 weeks) from May 2019 to January 2020. Following two cycles of CHOP chemotherapy, partial response (PR) was achieved based on the CT evaluation, and the largest lesion in the rectovesical pouch was 1.7*1.4 cm. (**Figure 1C**). Then, he accepted four additional cycles of CHOP. Due to the influence of coronavirus disease 2019 (COVID-19), he did not undergo imaging evaluation at the end of CHOP treatment. No serious nonhematological or late toxicities occurred during therapy. Unfortunately, only 2 months after receiving first-line chemotherapy, he had enlarged target nodal masses and newly occurring metastases in the mesentery by contrast-enhanced CT examinations in March 2020. The mass in the left midabdomen was 2.7 cm*2.8 cm (**Figure 1D1**), and the largest lesion in the rectovesical pouch was 2.0 cm*1.6 cm (**Figure 1D2**). Then, he achieved 2 cycles of ABVD chemotherapy (Adriamycin 25mg/m^2^ d1,15, Bleomycin 10mg/m^2^ d1,15, Vinblastine 6mg/m^2^ d1,15 and Dacarbazine 375mg/m^2^ d1,15 every 4 weeks) as a second-line treatment. Except for the mild nausea during the treatment, there was no other discomfort or obvious toxicity. However, the mass in the left midabdomen enlarged to 3.2 cm*3.0 cm, and the lesion in the rectovesical pouch increased to 2.0 cm*1.9 cm in June 2020 (**Figure 1E**). In June 2020, we administered to the patient a PD-1 inhibitor combined with an antiangiogenic agent: sintilimab 200 mg i.v. every three weeks and lenvatinib 14 mg orally once a day. After three cycles of treatment, the lesions size decreased (**Figure 1F**). After another three cycles of treatment, the mass lesion size continued to decrease, the mass in the left mid-abdomen shrank to 1.7 cm*2.2 cm (**Figure 1G1**), and the lesion in the rectovesical pouch also decreased in December 2020 (**Figure 1G2**). During the course of sintilimab combined with lenvatinib, the patients presented good tolerance to the combined approach. Myelosuppression, hypertension, diarrhea, or other adverse events, apart from fatigue, were not apparent. Disease developed again, as shown by a CT scan, in January 2021. PFS was 7 months following sintilimab and lenvatinib (**Figure 5**).

## Discussion

To our knowledge, this is the first report that provides a potential treatment option for FDCS patients with a combination of immune checkpoint inhibitors and antiangiogenic agents after the patient had progressed after chemotherapy.

The origin of follicular dendritic cells (FDCs) is uncertain. It is now generally believed that FDCS is caused by the abnormal proliferation and differentiation of follicular dendritic cells in the lymphoid follicle. Thus, FDCS is a malignancy of the hematopoietic system. Furthermore, previous studies suggested that although FDCs express phenotypic features of normal dendritic cells, the behavior is more akin to sarcoma rather than lymphoma (5). However, Krautler et al. revealed that FDCs arise from ubiquitous perivascular precursors (preFDCs) expressing platelet-derived growth factor receptor β (PDGFRβ). Their work highlighted that FDCs are stromal in origin rather than developing from mural cells (6).

FDCSs have an unclear etiology. Approximately 10% to 20% of FDCS cases are correlated with antecedent or concurrent Castleman disease, which is a benign lymphoproliferative disease, and mostly in the hyaline vascular variant (7). Two-thirds of FDC sarcomas occur in the lymph nodes, and one-third occur in extranodal sites (8). Most cases are asymptomatic, and lymphadenopathy is the most common clinical manifestation (7). The gold standard for the diagnosis is histopathological examination. Spindle cells can always be seen at the microscopic level. Immunohistochemical staining can distinguish FDCS from other spindle cell tumors. CD21, CD23 and CD35 could serve as markers of FDCS as they are usually positive in the majority of FDCS specimens (9, 10). In addition, clusterin was a sensitive and specific stain for FDCS. The rate of PD-L1 staining has been reported to be positive in 50%–80% of FDCS, which offers a rationale to use immunotherapy in patients with FDCS (11). Epidermal growth factor receptor (EGFR) is highly expressed in 100% of FDCS (12). Generally, the level of Ki-67 is approximately 20%, and positive Ki-67 expression is an important index of prognosis (13). Furthermore, PET/CT of our patient did not find focal hypermetabolism, although high-uptake regions were reported in other cases (11, 14).

There is no guideline for the treatment of FDCS. Surgical excision is the main treatment of choice for localized FDCS. The efficacy of adjuvant chemotherapy and radiotherapy is unclear. A pooled analysis of 462 patients with dendritic cell tumors revealed that adjuvant radiotherapy had no significant effect on the overall survival of patients (15). The study conducted by De Pas, T. et al. did not support adjuvant treatments after surgery (16). However, the importance of adjuvant treatment was suggested in another study (17, 18). For unresectable, recurrent and metastatic tumors, systemic chemotherapy, with or without radiotherapy, is often used to control symptoms. FDCS is frequently treated with lymphoma chemotherapy regimens such as ABVD and CHOP, although there is no consensus on the role of chemotherapy. In addition, gemcitabine combined with docetaxel was also used for the treatment, and such treatment yielded an overall response rate of 80% (19).

Recently, new drugs are deeply changing therapeutic standards of cancers such as immunotherapy and targeted therapy. But data is insufficient regarding the efficacy of such treatment modalities for FDCS given their rarity. Next-generation sequencing (NGS) was not performed in most cases and molecular aspects of this rare disease are still unknown. Pazopanib is an anti-angiogenic multi-targeted tyrosine kinase inhibitor used for treating soft tissue sarcomas and renal cell carcinoma (20). A patient received pazopanib after multiline therapy and had a partial response for 9 months (21). In addition, a patient with IHC for CD117 was positive showed complete pathological remission to the combination of imatinib (a BCR-ABL1 tyrosine kinase inhibitor), gemcitabine, and cisplatin (22). Immunotherapy also serves as a therapeutic option for such disease although the effect remains controversial. A Phase II clinical trial (NCT03316573) test the safety and effectiveness of Pembrolizumab, a PD-1 antibody, in treating aggressive lymphoma or a histiocyte or dendritic cell neoplasm including FDCS. The results have not been published (https://clinicaltrials.gov/ct2/show/NCT03316573). A case report showed that nivolumab was attempted to treat a patient with FDCS without any success (23). Another case report presented two recurrent patients with FDCS who were treated successfully with ipilimumab and nivolumab (11). These two patients showed stable disease after 8–12 weeks of starting therapy and showed marked improvement in symptoms.

However, those treatment options are limited. The median progression-free survival and overall survival times following frontline therapy were 21 and 50 months, respectively (18). The local recurrence rates range from 23% to 43%, and metastasis rates range from approximately 21%. The objective response rate to second- and third-line therapy was only 16%. Thus, developing a new treatment regimen is urgent.

ICI therapy, represented by anti–PD-1, has resulted in remarkable efficacy in the treatment of various hematological and solid metastatic malignancies (24–26). Tumor cells often express a programmed death ligand-1 (PD-L1), which binds to the programmed death receptor-1 (PD-1) on activated T-cells to induce immune tolerance (27). ICI therapy is deemed as the most promising approach for cancer control which changed the landscape of cancer therapies dramatically. However, durable antitumor responses are restricted to a minority of patients and the objective response rates of monotherapy were far from satisfactory in the clinical trials. Clarifying the mechanisms of tumor immune evasion and immune drug resistance are necessary for improving clinical outcomes for patients. Accumulating evidence has confirmed abnormalities in the tumor microenvironment (TME) were correlated with the efficacy of PD-1/PD-L1 blockade and the angiogenic factors could contribute to the immunosuppression in the TME by inducing vascular abnormalities, suppressing antigen presentation and immune effector cells (28–31).

Angiogenesis also plays a significant role in the pathogenesis of cancer, which could promote tumor proliferation and metastasis. Antiangiogenesis therapy is another promising strategy that has shown certain clinical responses in various solid cancers, such as colorectal cancer (32), hepatocellular carcinoma (33) and renal cell carcinoma (34, 35). However, the efficacy is still limited when used alone. Studies have shown that antiangiogenic agents have synergistic effects with PD-1/PD-L1 antibodies. Preclinical and clinical studies have confirmed the scientific rationale for the combination of PD-1/PD-L1 antibodies plus angiogenesis (36–38). This combination therapy could enhance T cell recruitment by normalizing tumor blood vessels (39). Tumor cells and vascular endothelial cells can release vascular endothelial growth factor (VEGF), which can promote tumor growth, invasion and metastasis and contribute to the immunosuppressive microenvironment (40–44). ICI combination with antiangiogenesis therapy has shown remarkable antitumor activity in multiple kinds of tumor types (45–49). For example, the results of IMBrave150 trial (50) and KEYNOTE-426 trial (51) demonstrated the survival advantage and safety of such combination therapy and thus changed the first-line treatment choice. However, no previous study has evaluated the efficacy of such combination therapy in FDCS.

The expression levels of PD-L1 were the most common biomarker of immunotherapy. Patients with PD-L1 overexpression are more likely to benefit from ICI. However, a subset of patients with high PD-L1 expression do not benefit from immunotherapy and PD-L1-negative patients also respond well to ICI (52, 53). Thus, predicting response remains challenging and PD-L1 expression alone was insufficient which restrain the application of ICI to some extent. Several potentially predictive biomarkers also have been studied which can be classified as tumor-related markers, tumor microenvironment-related biomarkers and host-related biomarkers (54–56). Tumor-related markers mainly including tumor mutational burden (TMB), microsatellite instability (MSI) and mismatch repair deficiency (MMR). Generally, high MSI is correlated with a stronger immune response (57) and patients with high-TMB are more likely to benefit from ICIs (58, 59). Infiltration of immune cells and the function of immune cells in the tumor microenvironment serve as prognostic factors in predicting the response (29, 60). Recent studies also indicated that the gut microbiome was associated with the efficacy of ICIs (61, 62). Further efforts are still required to translate whose markers into the clinical setting and to more accurately identify patients who will benefit from immunotherapy.

Considering the high expression of PD-L1 in our patient, the limitation of monotherapy and the synergistic effect of ICI combined with anti-angiogenesis therapy, such treatment regimen was given. Sintilimab, a fully human IgG4 monoclonal antibody which binds to PD‐1 to block the interaction of PD‐1 with PD‐L1 and PD‐L2, has been approved in China to treat patients with relapsed or refractory Hodgkin’s lymphoma (63). Lenvatinib is an oral multikinase antiangiogenic drug. During this combined treatment regimen, our patient did not complain any severe discomfort.

In conclusion, our case report provides clinical evidence of the efficacy and a manageable safety profile of a patient with advanced FDSC to the combination of immunotherapy and anti‐angiogenic agent, sintilimab plus lenvatinib, as subsequent‐line therapy. This case supplements the limited literature and provides a new therapeutic option for such rare disease. But our report also has limitations. For example, the PD-L1 expression is 90% in our case and the efficacy of monotherapy is unknown. Expanding the sample size and increasing the observation time are still needed in future research to verify its clinical application value. Additionally, due to multiple strategies could be used to treat such disease and the poor prognosis, a multidisciplinary evaluation is essential for diagnosing and determining an optimal treatment regimen for patients with FDCS. We also highlight the application of NGS technologies which may assist with future elucidation of effective treatments.

## Ethics Statement

Written informed consent was obtained from the patient for publication of this case report.

## Author Contributions

MJ treated the patient. YL wrote the case report. MJ and SZ reviewed in review the manuscript. All authors contributed to the article and approved the submitted version.

## Conflict of Interest

The authors declare that the research was conducted in the absence of any commercial or financial relationships that could be construed as a potential conflict of interest.

## Publisher’s Note

All claims expressed in this article are solely those of the authors and do not necessarily represent those of their affiliated organizations, or those of the publisher, the editors and the reviewers. Any product that may be evaluated in this article, or claim that may be made by its manufacturer, is not guaranteed or endorsed by the publisher.
